# Language practice and policy in Israeli hospitals: the case of the Hebrew and Arabic languages

**DOI:** 10.1186/s13584-019-0331-7

**Published:** 2019-07-02

**Authors:** Yael Keshet, Ariela Popper-Giveon

**Affiliations:** 1 Western Galilee Academic College, P.O. Box 2125, Akko 24121, The Max Stern Jezreel Valley College, Jezreel, Israel; 2 David Yellin Academic College, POB 3578, 91035 Jerusalem, Israel

**Keywords:** Multilingual organizations, Policy, Hospitals, Minorities, Israel

## Abstract

**Background:**

Organizational language practice and policy are not neutral elements but reflect social and political power relations. The micro-level of working groups is subject to the influence of political conflicts and power relations at the macro-level. In conflict zones in particular, these involve complex considerations. Consequently, the present research sought to examine tensions arising from the language spoken among mixed Jewish-Arab teams in Israeli public hospitals.

**Methods:**

In-depth interviews were conducted during 2016–2017, with 50 Jewish and Arab healthcare practitioners – 10 managers, 20 physicians, and 20 nurses – employed in 11 public hospitals in Israel.

**Results:**

Our interviews with healthcare practitioners revealed that speaking Arabic in the presence of the patient (not with the patient) may evoke negative feelings and resentment among both Jewish patients and colleagues. Moreover, conflicting attitudes may come into play when Arab practitioners speak Arabic among themselves. Two contexts of language use in Israeli public hospitals can be noticed: the language used in the presence of the patient; and the language used among the practitioners when no patient is present. The former involves the principles of cultural and linguistic competency, and is therefore governed by clear guidelines and procedures. The latter echoes the tensions between the two ethno-national groups in Israel, Jews and Arabs, and is not regulated by a clear policy formulated by the Ministry of Health or by the hospitals’ managements.

**Conclusions:**

Our analysis of language practice and policy as a multi-leveled phenomenon, where the micro-level of everyday interactions is influenced by the macro-level of political life, indicates a need for meso-level policy, led by the Ministry of Health. A policy of linguistic competency should be publicized and enforced to ensure that in the presence of the patient, practitioners speak a language s/he understands. This policy should also stipulate that among mixed teams of healthcare professionals every language is permissible, while the language spoken in a particular context should be understood by everyone present.

## Introduction

Sometime before we launched this research project, (the first author) recalls that while conducting participant observation in an operating room in a public hospital located in the North of Israel, she noticed a sign on the wall that read: “Hebrew only.” As a sociologist, the sign aroused her curiosity and she asked the head nurse of the operating room about it. She explained that the team that worked in the operation room comprised various practitioners, who spoke several languages: Hebrew, Arabic, Russian, and others. During surgery, the head nurse continued, some of the practitioners would at times begin to speak in a language that the rest of the team did not understand. Consequently, the hospital management decided that in the operating rooms only Hebrew – the dominant language in Israel spoken by all the employees – should be used.

The present article focuses on the experiences of healthcare practitioners employed in multilingual hospitals. It examines language practice and policy in Israeli public hospitals, which offer an appropriate site for researching the topic. Their medical staffs comprise both Jewish and Arab practitioners, who care for patients who belong to the Jewish majority and Arab minority groups in Israel, in the context of the ongoing violent national conflict between Israel and the Palestinians [1]. Tensions among medical staffs concerning language use may impair professional cooperation as well as practitioners’ linguistic competency, which refers both to their underlying ability and to language actual use during patient-practitioner interactions. As a result, such tensions may compromise the treatment of patients. Moreover, tensions pertaining to language tend to reveal as well as entrench power relations within healthcare organizations that may harm professionals, particularly those who belong to minority groups.

By studying the tensions arising from the language spoken among mixed Jewish-Arab teams in Israeli public hospitals, we sought to contribute to the scholarship on multilingualism in workplaces in general and in healthcare organizations in particular. We focus on the micro-level of the working group, where implicit policies merge with actual practices subject to the influence of political conflicts and power relations at the macro-level. We argue that meso-level policy, led by the Ministry of Health, is required to ensure competent healthcare and efficient teamwork.

### Language practice and policy in multilingual workplaces

The scholarship on multilingualism in the workplace addresses the manner in which organizations, such as large multinational firms and companies that operate in different geographical areas, handle linguistic diversity. Large-scale surveys were conducted to capture the language needs of different organizations [2]. While these studies reveal the multiplicity of languages that form the ecosystem of the various organizations, only a few companies, predominantly the larger ones, had clear strategies in place to meet those needs [3].

Language policy can be understood as the explicit and implicit policies used in an attempt to change the language behavior of individuals within a society [4]. Language policy lays out language management – the facilitation and coordination of communication between members of different speech communities [5]. Language policies can be conceptualized and studied as multi-leveled phenomena [6] that span different levels of management on a continuum from a “macro” supranational or national governmental level, through a “meso” organizational level, to a “micro” working group level, where it might be difficult to distinguish between implicit policies and the actual practices [3]. As scholars have argued [7, 8], ambiguity with regard to language policy is common; guidelines may overlap and intersect and companies may promote a particular corporate language while permitting the use of other languages if this furthers their economic interests.

While organizations frequently present the imposition of a one language policy as a “neutral” action, this is often an ideological decision that specifically impacts the management of the power (im)balance between individuals, teams, or departments. An organization’s choice of language is directly related to its social, political, and moral order, since some languages enjoy a higher status than others [3]. The choice of language in the organization may thus be made to include and/or exclude others from the various encounters that occur in the workplace. As values and beliefs are associated with language use [9], they become central in understanding both policy and practice, particularly with regard to decisions made by those in power [3].

Researching organizations’ choice of language requires an understanding of the negotiation of identity, expertise, power, and status [10]. Thus, a critical view of multilingualism and language policy and practice must look beyond the language policy itself as a set of distinct and concrete rules.

Healthcare organizations, particularly those that operate in multicultural and multilingual contexts, consider mostly the various implications of language use for patients. Effective communication between patients and clinicians was found to be a critical component of high-quality healthcare [11]. Offering language support to linguistic minorities, in particular, can improve patient safety, clinical outcomes, and the quality of healthcare [12]. Language barriers, on the other hand, have been shown to constitute a major cause of healthcare disparities [13, 14]. Ensuring that patients belonging to linguistic minorities have the means to communicate effectively with their healthcare providers is therefore critical to improving their experience in the healthcare setting, the quality of care they receive, and their health outcomes [11].

As racial and ethnic diversity in many countries worldwide increases, hospitals are required to provide language services that meet the needs of people in their communities [15]. Enhancing the racial and ethnic diversity of the healthcare workforce is one of the means essential to the adequate provision of linguistically competent care to minority communities [16, 17]. This measure furthermore plays an important role in reducing health disparities among different ethnic groups in the population [18, 19].

Extensive scholarship has addressed the positive outcomes associated with language competency in healthcare organizations, and specifically with the role that ethnically diverse teams can play to promote effective communication between patients and clinicians (e.g., [16, 17, 19]. These studies have, regrettably, focused almost exclusively on *patient-practitioner* relations and the policy of cultural competency that relates to it. We know of no study that has dealt with the complexities of multi-linguistic *teams* in healthcare organizations. As mentioned above, language plays a primary role as a determinant of social identities and work relations in multilingual hospitals. In practical terms, there is a need to formulate an equitable language policy that recognizes and respects cultural diversity in healthcare organizations [20].

### The Israeli context

Israeli society comprises a diversity of cultural groups that differ in religion, origin, area of residence, level of religiosity, and other characteristics. Linguistic differences are clearly evident in Israel, and to a large extent overlap the cultural map of the country’s citizens. Despite the diversity of languages spoken in Israeli society, in this article we focus on Hebrew and Arabic. Hebrew is the majority language in Israel, spoken by the majority Jewish population (74.7%). The largest linguistic minority in Israel is constituted by speakers of Arabic. Today, about one-fifth of Israel’s population (20.9%) are Arabs who speak Arabic as their mother tongue [21].

Prior to 2018, Arabic was an official language in Israel, alongside Hebrew. On March 13, 2018, a bill was tabled in parliament that stipulates, inter alia, that the status of Arabic be downgraded from an “official language” to that of “a language of special status,” merely in order to further Arabic speakers’ linguistic accessibility to state-provided services [22]. This bill became a basic law of Israel on July 19, 2018. The new “Basic Law: Israel – The Nation State of the Jewish People” [23] downgrades the status of Arabic by making Hebrew the only official national language, while Arabic has been demoted from an official language to a language of “special status.” This was part of a broader shift by which Israel was declared to be the nation-state of the Jewish people and which transcended the issue of language. Some regard this initiative as a violation of the basic rights of the Arab ethno-national minority in Israel. The Arabic language is part of the identity, heritage, and culture of the Arab minority, and the recognition of Arabic as an official language therefore symbolizes the recognition of the Arabs’ rights and equal status in the state of Israel [24].

Every Israeli resident is entitled to health services under the National Health Insurance Law [25]. Nevertheless, although both Jews and Arabs are treated in Israel’s public healthcare system, there is no explicit law regulating the linguistic accessibility of healthcare services to the country’s various population groups. However, legislation has established certain principles that may have implications for various aspects of linguistic accessibility in the public healthcare system. The Patients’ Rights Law [26], for example, stipulates that medical treatment shall not be administered to a patient unless he or she has given their informed consent. In order to obtain such informed consent, the clinician shall provide patients with the medical information they need, in a reasonable manner, in order to enable them to decide whether they agree to receive the proposed treatment. Hence, this medical information must be communicated in a language that the patient understands.

In 2011, the Israeli Ministry of Health issued a Director General’s Circular entitled “Cultural and linguistic adaptation and accessibility in the healthcare system” [27]. The circular recognizes that the heterogeneity of Israeli society in terms of religion, culture, and language may affect health and illness, the use of healthcare services, morbidity rates and patterns, and a number of other health indicators. The circular was aimed at ensuring that non-Hebrew speakers receive adequate medical services and at reducing health disparities among Israel’s various population groups. It stipulates that administrative materials, such as forms, various medical documents, and internet sites used by patients should be written in four languages: Hebrew, Arabic, Russian, and English. Public healthcare organizations are required to operate service centers and public information bureaus in Hebrew, Arabic, Russian, English, and Amharic (an Ethiopian language). Forms that patients are asked to sign as well as the various signs posted within healthcare organizations should also be comprehensible to those who do not read Hebrew.

A recent study [28] reviewed the actual cultural and linguistic accessibility of 35 general hospitals in Israel (out of the 39 general hospitals that were then). The study found that prior to the introduction of the Ministry of Health Director General’s Circular on cultural and linguistic adaptation and accessibility in the health system, the level of cultural competence in the hospitals was not high. Only about 22% of the signs in the hospitals examined met the requirements of the circular (i.e., signs in Hebrew, Arabic and English). Another report [29] states that various health organizations use different means of linguistic accessibility and cultural adaptation. The circular is applied unevenly in various organizations, and it seems that only a minority of health workers have been trained for cultural competence.

One of the effective means of enhancing cultural and linguistic competency in Israeli healthcare organizations is to recruit Arab healthcare professionals. In Israel, a relatively high percentage of Arabs is employed in the healthcare professions [30, 31]. A career in medicine or nursing provides a stable income and enables Arab citizens to integrate in the (Jewish dominated) Israeli labor market. Medicine in particular is perceived to offer a pathway to individual excellence and a means toward achieving socioeconomic mobility [30]. Arabs working in healthcare organizations, especially in public hospitals, regard them as enclaves in which the differences between the two ethnic groups – Jews and Arabs – become less acute as practitioners focus on the universal needs of the human body, and on questions of illness and health, life and death. Arab practitioners view healthcare organizations as a relatively egalitarian setting in which they are accepted as equals; as a humanistic and apolitical environment in which the hostility between Jews and Arabs is mitigated and relationships conform to the “one big happy family” ideal [32].

Alongside the integration into Israeli society that employment in the healthcare system offers Arab citizens in Israel, working shoulder to shoulder with Jewish colleagues generates tension regarding language practice and policy among the mixed Jewish and Arab teams that operate in the country’s healthcare organizations, which is exacerbated by the ongoing violent national conflict between Israel and the Palestinians. The present study thus sought to examine tensions that arise from the issue of the language used among mixed teams in Israeli public hospitals. Its primary objective was to document and to analyze situations in which tensions arise concerning the use of Arabic or Hebrew in order to propose an appropriate policy to handle such situations.

## Methodology

We conducted a comprehensive qualitative study among Jewish and Arab healthcare practitioners employed in 11 public hospitals in Israel. A total of 50 in-depth interviews were conducted during 2016–2017, with ten managers (senior nurses, department heads, etc.) – seven of whom were Jewish and three were Arabs, and with 40 physicians and nurses – ten Jewish physicians, ten Arab physicians, ten Jewish nurses, and ten Arab nurses – employed at eleven public hospitals in Israel. We used a snowball sampling, a method employed extensively in studies dealing with sensitive matters [33]. We preferred reaching out from one participant to another on a personal basis and not through the administration of the facilities at which said participants are employed. We were thus able to guarantee greater anonymity and alleviate fears of expressing one’s views and experiences. Researchers who use this form of sampling initially select a few participants (a convenience sample) and ask them to recommend others who meet the designated criteria (in our case, employed as a manager, a physician, or a nurse in an Israeli public hospital) and who would be interested in participating in the study. Since such recruitment could limit heterogeneity among participants, we made sure to interview practitioners from a wide range of large public hospitals situated in various parts of Israel.

The 30 to 90-min long interviews were conducted in Hebrew, a language all participants speak fluently. The second author and a research assistant conducted the interviews. Participants were asked open-ended questions regarding the relationships between Jews and Arabs at their respective hospitals; how the realities of life in Israel affect working relations at healthcare organizations; whether a policy is in place regarding these issues and if not, what kind of policy they would recommend.

No incentives were offered the participants. After securing oral permission from participants, interviews were tape-recorded and transcribed verbatim. We used conventional qualitative content analysis [34], adopting inductive reasoning, through which themes and categories emerge from the raw data under the researcher’s careful examination and constant comparison [35].

The interview transcripts were analyzed using Atlas.ti v7.5.17 textual analysis software for systematic coding and inductive analysis. ATLAS.ti was employed to support two processes – data management and coding. Data management refers to the process of managing the large set of data records collected during the interviews, while data analysis refers to the process of coding these materials. The ATLAS.ti software enabled us to develop a coding schema that indicated the topics or concepts that emerged from the data. This involved selecting quotations and assigning them a code, after which all quotations assigned the same code were retrieved by running a report, or viewed in context by using the code manager. This process facilitates retrieval of related quotations in order to examine patterns and trends in the data, and facilitates the grouping of codes into categories that represent broader and more abstract themes. The study was financed by the Israel National Institute for Health Policy Research and approved by the ethics committee of the Western Galilee Academic College.

## Results

The interviews conducted with Jewish and Arab healthcare practitioners employed in Israeli public hospitals revealed two contexts of language use in healthcare organizations, around which the following section is constructed: 1. the language spoken with the patient and in the patient’s presence; 2. the language spoken among the practitioners when no patient is present. The former touches on the principles of cultural and linguistic competency, and is therefore articulated in clear guidelines and procedures [36]. The latter context is bound up with national tensions and feelings of hostility and even fear, which manifest the distance between Israel’s two ethno-national groups, Jews and Arabs. By the time the interviews were conducted, neither the Ministry of Health nor the hospitals’ managements has issued clear guidelines to regulate the use of language among practitioners.

### Talking with the patient and in the patient’s presence

We found a broad consensus among interviewees that the language used in their communication with the patient or in his or her presence should, if possible, be understood by the patient. All the Arab healthcare practitioners employed in Israeli public hospitals speak Hebrew fluently. Most Jewish patients, on the other hand, do not understand Arabic. Hence, interviewees agreed that only Hebrew should be spoken in the presence of a Jewish patient, whereas if everyone present, including the patient, speaks Arabic, then the conversation may be held in Arabic.

During rounds… If there are three young doctors and an intern, and the intern speaks Arabic and so does another doctor, and they come to a family that speaks Arabic, the conversation will be in Arabic (Jewish physician)

All the interviewees agree that the patient should understand the language used by the attending practitioners. In other words, if the patient is Arab and the practitioners are Jewish, either the practitioners should learn some Arabic or someone has to translate.

We need to communicate with the patients somehow... Most practitioners at [hospital name] can take a basic anamnesis and understand what the medical problem of a patient is in Arabic… they have learned from experience, pick up a word here and a word there (Jewish physician)

When I worked in pediatric intensive care, there were ten beds. Usually, eight out of the ten were Arab patients. But there are almost no Arab professionals in that ward... There is an Arab guy and he speaks Arabic with them. He explains everything to them, it also gives them confidence... Usually what I do, I say 'Mahmoud, can you come and translate?' (Jewish nurse)

However, sometimes, such as during rounds or the switching of shifts, interviews reveal that the practitioners do not talk *to the patient* but *about the patient*. The interviews reveal that practitioners are aware of the importance of using a language understood by patients, even when they speak to one another in their presence and not directly to them.

At the patient's bedside you have to speak the language that the patient understands. I don’t agree with those who speak their language while the patient is sitting there like an idiot. Among ourselves, as we sit and drink coffee, we can speak any language, whether it’s Russian, German, English, or Arabic. But at the patient's bedside it is desirable, even the hospital management requests this, to speak a language that the patient understands (Arab nurse)

In other words, the practitioners interviewed agree that in the patient’s presence they ought to speak a language the patient understands, especially when the patient is Jewish, since all Arab practitioners speak Hebrew fluently, whereas the vast majority of Jewish patients does not understand Arabic.

If a patient is a Hebrew speaker and all the practitioners present are Arabs and they speak Arabic over his head and he does not understand, I think this is problematic... What matters is that the patient understands, that he doesn’t feel the practitioners are arrogant (Jewish manager)

During breaks or in the cloakroom, we can speak Arabic. But on the ward, whenever we discuss patients' issues, we cannot speak Arabic... After all, we live in the State of Israel and we must be able to speak Hebrew (Arab nurse)

Notwithstanding this consensus, some Jewish interviewees described situations in which Arab practitioners spoke Arabic in the presence of a Jewish patient, even when the latter did not understand them.

It may happen that the doctor is an Arab, the nurse is an Arab, and the patient and the family are Jewish. The doctor and the nurse begin to speak Arabic, as the patient lies there and doesn’t understand what they are talking about. Is this okay? I don’t know. I don't think so (Jewish nurse)

Such a situation, in which the practitioners talk *about* the patient in his/her presence but do not *address him/her directly*, can occur, for example as the interviews reveal, during the switching of shifts or during rounds:

I was present once on a round, as a senior nurse. They [the doctors] were consulting in Arabic regarding the patient, who was a Hebrew speaker. The Arab neurosurgeon and the Arab nurse answered the doctor and the nurse in Arabic. No one spoke Hebrew. And then I said “friends... Arabic doesn’t bother me, it bothers me that the patient doesn’t understand what you are saying, and that I don’t understand what you are saying. And since you all speak Hebrew, you should switch to Hebrew now” (Jewish nurse)

Some Jewish interviewees noted that the sound of the Arabic language within the hospital evokes strong emotions among some of their Jewish colleagues. For example, a Jewish nurse recounted an experience, relayed to her by one of her colleagues, in an emergency room in a different hospital, where the Arab practitioners spoke Arabic. Her colleague said that she felt that she was not in Israel but in the Palestinian Authority; that the situation echoed the violent national conflict in the region.

My deputy was in an emergency room at [name of] hospital because her son fell and had a cut. She came back in a state of shock. I asked her “Why? what happened?” She said “Don’t ask, an Arab doctor, an Arab nurse, all of them were Arabs and they spoke only Arabic. I felt as if I were entering a hospital in Nablus [a city in the West Bank, associated as an important political, commercial and cultural Palestinian center]”... She had a shocking, terrible experience, she says. She told me she was too embarrassed to ask them to speak Hebrew so that she could understand them (Jewish nurse)

By contrast to the unease caused by the use of Arabic in the presence of a Jewish patient, the reverse situation, namely the use of Hebrew among practitioners in the presence of an Arab patient who does not understand Hebrew, was hardly mentioned in interviews as a source of resentment. It seems that Arab patients who come to be treated in public hospitals in Israel, belonging a minority population, assume that the language spoken in the hospital by practitioners will be Hebrew. The practitioners speak Hebrew in the presence of Arab patients who do not speak Hebrew as a matter of course, and call in a translator when speaking with them.

There is always someone that knows Arabic in the room or the next room, and you can always ask someone to come and translate... but the official language is Hebrew... Everything is conducted in Hebrew… Every hospital in which I worked used one language. French in France… All kinds of people speak all kinds of languages, but in Israel… this is all conducted in Hebrew (Jewish physician)

Unlike Arab patients, who expect to hear the Hebrew language, the interviewees report that for some Jewish patients the very sound of the Arabic language in the hospital is disturbing, and they complain. This is one of the reasons why in some hospitals and wards practitioners are instructed to speak only Hebrew, unless the patient is Arab and the practitioners are therefore required to act in the spirit of cultural and linguistic competency and use Arabic when they communicate with him or her.

The patients complain, sometimes the staff members complain. Therefore, the instruction is that one must speak Hebrew (Arab manager)

However, the language policy in Israeli public hospitals is not clear to the interviewees, and many interviewees cannot recall precisely what is stipulated and where.

If they [the Arab practitioners] are sitting in the staffroom and they are the only ones there, that's okay. They can speak their own language. But if there are other staff members or patients present, they should speak Hebrew... I don’t remember if this is written, but it was decided upon. I mean, yes, I think they issued an instruction... In the Nursing Administration (Jewish manager)

There is no instruction regarding the language spoken in the organization but there are instructions regarding the language spoken with the patient. This is described as part of the respect shown to the patient or the person you are addressing. If you speak a language – speak a language that the other person understands (Arab manager)

The interviews indicate that while an effort is made to communicate with patients in a language they understand, when colleagues speak to one another in the patient’s presence (and not to them directly) this rule is not always adhered to. Furthermore, it is difficult to enforce a “Hebrew only” policy in the wards, since this may be perceived as an act of discrimination by the Arab practitioners. The heads of wards and hospital directors thus find their own way of coping with this sensitive issue:

[At the time] quite a few Arab doctors joined my department and they spoke Arabic, even during morning rounds or afternoon staff meetings. I was very upset, but on the other hand I didn’t want to comment because I was afraid that it would insult them and I didn’t want to disturb the atmosphere... I asked one of the senior Arab doctors at some stage, when I realized the situation was getting out of hand, that at the patients’ bedside they speak Arabic and people don’t understand... this was not professionally appropriate. I approached this doctor in private, and I told him, and shared my dilemma. And he himself told me that it bothered him too... Actually he said he would take care of it. Since then, whenever they speak Arabic, he switches the language (Jewish manager)

There is no such thing as language policy. The only thing that counts is the actual practice in the field. About a year and a half ago, there was an incident in [name of hospital], whose manager ordered the teams not to speak Arabic among themselves over the bed of a patient who doesn’t understand Arabic. And this became a big issue. Some perceived it as an act that considered the patient's needs and others perceived it as a racist statement (Jewish manager).

To sum up, the interviewees describe Israeli public hospitals as places that promote culturally and linguistically competent treatment. Consequently, when the patient speaks Arabic the staff tries to ensure that the communication will be in Arabic, even if this requires a member of staff or a relative to act as a translator. However, according to the interviewees, Arab practitioners sometimes speak Arabic in the presence of patients who do not understand the language, for example during a change of shifts or on rounds. This practice arouses negative emotions and resentment among both Jewish patients and practitioners. Thus, while a policy of speaking *with the patient* in a language the patient understands is emphasized and enforced, as in the context of existing legislation that requires patients to give their informed consent to medical treatment, no clear policy is in place on *speaking about the patient* in the patient’s presence. The organizational guidelines on this issue are vague and there are evident impediments to enforcing them.

### The language used among practitioners

Alongside the question of the language spoken with patients or in their presence and its implications for cultural and linguistic competency, the issue of the use of Arabic among the practitioners themselves when no patient is present was raised in the interviews. This concern does not necessarily address the treatment of the patient, and is therefore irrelevant to the principles of cultural and linguistic competency. Rather, it resonates with the relations between the Jewish majority and the Arab minority in the country.

The use of Arabic among staff members when no patient is present covers both private conversations and professional communications. The interviews point to three types of situation: situations when no Jewish professionals are present; situations when Jewish professionals are present, but who just happen to be there and are not party to the conversation; and situations when Jewish professionals are present and take part in the conversation. This ambiguity is not currently addressed by hospital guidelines nor in the policy directives issued by the Israeli Ministry of Health.

Interviews indicate that Arab practitioners sometimes speak Arabic among themselves in the hospitals:

If you and I are somewhere near a patient and the patient speaks Hebrew and doesn’t understand Arabic... we are supposed to speak Hebrew... but if you and I are alone and there is no-one else, then we speak the language we want (Arab manager)

I think it would improve the treatment if two Arabic-speaking doctors decide to discuss the patient in Arabic. It is preferable that they communicate the information in Arabic. Moreover, Arabic is an official language in the country, there is no reason not to use it (Arab physician)^1^

Despite the common understanding that in healthcare organizations it is necessary to use a language that everyone understands, in practice situations occur when Arab practitioners speak Arabic among themselves in the presence of a Jewish colleague who does not understand Arabic. These situations arouse antagonism and tension.

When doctors hold some discussion among themselves, if they speak Arabic how can I take part in the discussion? I don’t understand... In a situation like this, sometimes you comment, sometimes you don’t, sometimes you get angry, sometimes there are conflicts. Sometimes you comment politely, sometimes you make a rude remark. It depends (Jewish nurse)

At times Jewish practitioners will comment to their Arab colleagues on the use of Arabic in the organization, generating anger and resentment among the latter.

There were two practitioners who spoke Arabic among themselves, maybe even a little louder. And there was this nurse, who they felt was always gunning for them, I mean, just waiting for them to speak Arabic so that she could rebuke them (Jewish manager)

Once, there was some kind of ethnic conflict. The head of the department talked to the interns, who spoke Arabic in the operating room. She told them, “Here we speak only Hebrew” and it raised a fuss... She took it to the management, just like that, she is a pretty strong woman, and said that this should be stopped... I heard the Arab interns talk about feeling anger, injustice... They felt it was arrogant (Jewish manager)

The interviews reveal that in the absence of guidelines issued by the Ministry of Health regarding the language spoken by practitioners when patients are not present, the heads of nursing or heads of department in certain hospitals have drawn up such guidelines. It is, however, sometimes difficult to distinguish between implicit policies and actual practices, and this vagueness is reflected in the interviewees’ responses.

There are instructions, I don’t know if it's a policy or just something written in the hospital’s "white paper"... It's a request of sorts to try to speak Hebrew. It is the official state language (Arab manager)

I haven’t seen it [the instruction to speak Hebrew] in writing. But as soon as they recruited me as a nurse, during my overlap with the other nurses, I did hear about it. I didn’t even know at first. I started to speak Arabic and the head nurse took me aside and told me, “We speak only Hebrew here.” (Arab nurse)

Besides interviewees who mention specific guidelines that instruct members of staff to speak Hebrew in certain wards or hospitals, others maintain that there is no clear policy in Israeli healthcare organizations regarding this issue. Since the question of the language spoken among practitioners (when no patient is present) is not a matter of cultural and linguistic competency but rather an issue of majority-minority relations, many hospital directors, fearful of hurting employees’ feelings and igniting disputes, prefer not to deal with this “hot potato.” As the interviews reveal, in such organizations practitioners are expected to exert self-control and to make sure they speak the majority language, which everyone understands.

There is no such a policy... I think that there should be a policy regarding this matter but it is problematic to publicize it because people might feel hurt… I would like people to understand this on their own... I think it's a *chutzpah* [to converse in a language] that not all those present in the room understand. It's an impolite act ... You are in a work environment; everyone hears, everyone responds, everyone works together, there is a certain rhythm. If someone is out of this rhythm - it disturbs the harmony (Jewish manager)

I don’t think that this is a policy or a law, but we are committed to the patients; to speak a language that the patient can understand. Practitioners, among themselves, I think this is a matter of respect, not of procedure (Arab nurse)

## Discussion

The present research sought to examine tensions arising from the language spoken among mixed Jewish-Arab teams in Israeli public hospitals. This appears to be an innovative study since we know of no study to date that has addressed the complexities of multi-linguistic teams in healthcare organizations, particularly in conflict zones. The interviews reveal two main aspects of language use in Israeli public hospitals. One aspect is the language used to communicate with patients or spoken in their presence; and the second is the language used among practitioners when no patient is present. According to the interviewees, Israeli hospitals promote culturally and linguistically competent healthcare, and practitioners strive to communicate with patients in a language they understand or else use interpreters, since they are aware of the policy on this matter. However, no clear policy is in place concerning the language spoken among practitioners in the presence of the patient – such as during a change of shifts or on the physicians’ rounds in the wards. Speaking Arabic in these contexts may evoke negative feelings and resentment among both Jewish patients and colleagues.

Conflicting attitudes may also come into play when Arab practitioners speak Arabic among themselves. The resulting reactions echo the political conflict between Israel and the Palestinians and the ethno-national power relations between Jews and Arabs within Israeli society. Jewish participants reported that Arab practitioners sometimes spoke Arabic in the presence of Jewish staff who did not speak the language, arousing tensions, antagonism and anger in Jewish staff. When Jewish practitioners reprimand their Arab colleagues, this in turn generates resentment among the latter. Certain hospitals or wards have apparently issued guidelines that require their staff to speak only Hebrew, yet in many cases it is difficult to distinguish between explicit or implicit policy and actual practices.

The impact of the conflictual political power relations at the macro-level of society on the micro-level of working group interactions is revealed in the interviews. Ethno-national conflicts within Israel, for example over the status of the Arabic language, as well as the longstanding conflict between Israel and the Palestinian authority and some neighboring Arab countries, permeate the hospitals and are reflected in the tension surrounding the issue of the language spoken with patients and within the mixed teams. These conflicts have surfaced lately in the context of the new “Basic Law: Israel - The Nation State of the Jewish People” (2018), which made a far-reaching impact on Israel’s minorities. The law downgrades the status of Arabic by making Hebrew the only official national language. With this law, Arabic has been demoted in importance from an official language to a language of “special status.”

However, linguistically competent treatment in healthcare organizations is a medical necessity. Thus, in 2011 The Israeli Ministry of Health formulated a policy with respect to linguistically competent treatment, which stresses the importance of speaking a language that the patient understands [27]. This is, however, not the case with respect to practitioners who converse among themselves, when no patient is present. The absence of a policy on the issue creates a vacuum in which macro-level tensions permeate the micro-level interactions. We suggest that the introduction of a sensitive policy by the Israeli Ministry of Health and its enforcement by hospitals’ managements could moderate the effects of power relations at the macro-level on the micro-level tensions among mixed teams.

What should such a policy statement contain? Policy at the meso-level initiated by the Ministry of Health should address both contexts of language use in healthcare organizations. The first concerns the language spoken with patients and in their presence. This aspect is related to cultural and linguistic competency, and hence also to the aspiration to reduce healthcare disparities between different population groups. This policy should be better implemented, both by the Ministry of Health and by hospital managements. The second context concerns the language spoken by practitioners among themselves, beyond the patients’ earshot. These include situations when no Jewish professionals are present; situations when Jewish professionals happen to be present but are not party to the conversation; and situations when Jewish professionals are present and take part in the conversation. Clear policy on this issue is essential in order to reduce tension among the staff and thereby facilitate optimal teamwork. This is a complex issue since, as a rule, certain languages ​​enjoy a higher status than others in a particular society [3]. In Israel, given the conflictual nature of relations between Jews and Arabs, this is a particularly sensitive issue on which no clear guidelines have been laid down and which is consequently shrouded in ambiguity.

The present research has several limitations. Its objectivity might be compromised, although the interviews were coded by both authors and the system of categorization was discussed several times in order to minimize bias. Furthermore, given the retrospective design of the study, a memory bias cannot be ruled out. The sample might also be prone to selection bias, as we provide data collected from 50 interviewees who agreed to participate. The decision regarding whom to contact may have been biased by the researchers and research assistant’s familiarity with some of the interviewees. With snowball sampling on politically sensitive subjects, in particular, there is a serious concern that the initial interviewees will share the political orientations of the authors, as will subsequent interviewees recommended by the initial interviewees. Moreover, the study did not include interviews of patients, and thus could not ascertain directly how patients feel about the use of a language they do not understand by professionals conversing among themselves. In sum, with 50 respondents overall, we cannot estimate the prevalence and impact of the challenges and problems identified, but only identify key challenges, begin to understand them, and engage in discussions about how they should be addressed. Future research based on an observational study should provide a complementary and more profound analysis of the subject.

## Conclusions

A policy introduced at the meso-level, initiated and enforced by the hospital management, could help reduce tensions at the micro-level. Such a policy must take account of the power relations manifested in the language used, of the marginalization of certain languages, and of the sense of exclusion on the part of practitioners who do not understand a certain language. The implementation of a carefully crafted policy could help reduce tensions surrounding this issue in healthcare organizations.

Our recommendations are as follows: First, the policy of linguistic competency should be publicized and enforced in public hospitals to ensure that in the presence of the patient, practitioners speak a language s/he understands. Second, a policy should be introduced stipulating that among mixed teams of healthcare professionals every language is permissible, but that emphasizes that the language spoken in a particular context should be understood by everyone present. Such a policy would ensure that Hebrew speaking practitioners understand all the communication on the team, while allowing Arab practitioners to speak their language among themselves. By establishing a balance between inclusion and exclusion, and between formal regulations and practice, such a policy could help reduce tensions between minority and majority healthcare practitioners in public hospitals.

The Israeli Ministry of Health [37] has recently issued a specific directive, declaring that the use of “foreign” (non-Hebrew) languages (and Arabic in particular) in healthcare organizations cannot be prohibited. Exceptions to this ruling apply to the use of the “foreign” language during routine work, such as on rounds, and at staff meetings, and when a language that is not spoken by the entire team or by the patients is used in a way that compromises medical competence. The directive specifies that interaction between practitioner and patient should be conducted in a language understood by the patient. However, if the patient, the practitioner, and all the other staff members, without exception, speak a language other than Hebrew and wish to converse in this language, this is permitted and even desirable.

Tensions around the use of languages other than Hebrew in healthcare organizations in Israel is particularly evident in the case of Arabic, which evokes negative feelings among some Jews in the context of the ongoing violent national conflict between Israel and the Palestinians. Some tensions are, however, evident with regard to the Russian language as well, which is widely spoken in Israeli hospitals since the wave of Jewish immigrants who arrived in Israel from the former Soviet Union in the 1990s, among whom were many healthcare practitioners. Future research is therefore recommended regarding the use of the Russian language in Israeli healthcare organizations.

## Data Availability

Not applicable.

## Footnotes

1 The interview was held before the new law was passed downgrading the status of the Arabic language.
